# The Effect of Pulmonary Artery Catheter Use on Costs and Long-Term Outcomes of Acute Lung Injury

**DOI:** 10.1371/journal.pone.0022512

**Published:** 2011-07-21

**Authors:** Gilles Clermont, Lan Kong, Lisa A. Weissfeld, Judith R. Lave, Gordon D. Rubenfeld, Mark S. Roberts, Alfred F. Connors, Gordon R. Bernard, B. Taylor Thompson, Arthur P. Wheeler, Derek C. Angus, for the NHLBI ARDS Clinical Trials Network

**Affiliations:** 1 CRISMA (Clinical Research, Investigation, and Systems Modeling of Acute Illness) Center, Department of Critical Care Medicine, University of Pittsburgh, Pittsburgh, Pennsylvania, United States of America; 2 Department of Biostatistics, University of Pittsburgh, Pittsburgh, Pennsylvania, United States of America; 3 Department of Health Policy & Management, University of Pittsburgh, Pittsburgh, Pennsylvania, United States of America; 4 Department of Medicine, Sunnybrook Health Sciences Centre, Toronto, Ontario, Canada; 5 Departments of Medicine and Industrial Engineering, University of Pittsburgh, Pittsburgh Pennsylvania, United States of America; 6 Department of Medicine, Case Western Reserve University, Cleveland, Ohio, United States of America; 7 Department of Medicine, Vanderbilt University, Nashville, Tennessee, United States of America; 8 Department of Medicine, Harvard University, Boston, Massachusetts, United States of America; Universidad Peruana Cayetano Heredia, Peru

## Abstract

**Background:**

The pulmonary artery catheter (PAC) remains widely used in acute lung injury (ALI) despite known complications and little evidence of improved short-term mortality. Concurrent with NHLBI ARDS Clinical Trials Network Fluid and Catheters Treatment Trial (FACTT), we conducted a prospectively-defined comparison of healthcare costs and long-term outcomes for care with a PAC vs. central venous catheter (CVC). We explored if use of the PAC in ALI is justified by a beneficial cost-effectiveness profile.

**Methods:**

We obtained detailed bills for the initial hospitalization. We interviewed survivors using the Health Utilities Index Mark 2 questionnaire at 2, 6, 9 and 12 m to determine quality of life (QOL) and post-discharge resource use. Outcomes beyond 12 m were estimated from federal databases. Incremental costs and outcomes were generated using MonteCarlo simulation.

**Results:**

Of 1001 subjects enrolled in FACTT, 774 (86%) were eligible for long-term follow-up and 655 (85%) consented. Hospital costs were similar for the PAC and CVC groups ($96.8k vs. $89.2k, p = 0.38). Post-discharge to 12 m costs were higher for PAC subjects ($61.1k vs. 45.4k, p = 0.03). One-year mortality and QOL among survivors were similar in PAC and CVC groups (mortality: 35.6% vs. 31.9%, p = 0.33; QOL [scale: 0–1]: 0.61 vs. 0.66, p = 0.49). MonteCarlo simulation showed PAC use had a 75.2% probability of being more expensive and less effective (mean cost increase of $14.4k and mean loss of 0.3 quality-adjusted life years (QALYs)) and a 94.2% probability of being higher than the $100k/QALY willingness-to-pay threshold.

**Conclusion:**

PAC use increased costs with no patient benefit and thus appears unjustified for routine use in ALI.

**Trial Registration:**

www.clinicaltrials.gov NCT00234767

## Introduction

The National Heart, Lung and Blood Institute (NHLBI) Acute Respiratory Distress Syndrome (ARDS) Network recently reported the results of the Fluid and Catheter Treatment Trial (FACTT), a multicenter study that simultaneously randomized subjects with acute lung injury (ALI) to one of two fluid management strategies (liberal or conservative fluid administration) guided by one of two hemodynamic monitoring devices (a pulmonary artery catheter (PAC) or a central venous catheter (CVC)). FACTT failed to demonstrate that PAC-guided fluid management of patients with ALI improved hospital outcome.[1] Other studies have also been unable to show improved outcomes through use of the PAC and some have attributed harm to its use.[2]–[4]

Nonetheless, recent evidence suggests several hundred thousand Americans receive PAC-guided care each year for ALI and other conditions.[5] An argument in favor of continued PAC use is that the increased physiologic data available to clinicians facilitates clinical decision-making, which may reduce time in the intensive care unit (ICU), time requiring mechanical ventilation, and the considerable hospital and post-discharge morbidity and healthcare costs of ALI.[6]–[9] Therefore, despite lack of evidence in improving short-term outcomes in critically ill patients with ALI, the PAC could be associated with decreased short and long-term resource use.

## Materials and Methods

### Objective

We hypothesized that the PAC could have long-term economic impacts not previously evaluated in a recently published multicenter short-term efficacy trial. Concurrent with the FACTT trial, we conducted a prospective economic assessment of the PAC (EA-PAC study) out to one year after hospital discharge to determine the clinical and economic consequences of using the PAC in the management of ALI.[10]

### Study design and participants

Details of the study design for the underlying ‘parent’ trial, FACTT, were published previously.[1] Briefly, FACTT was a 2×2 factorial design multicenter trial conducted by the NHLBI ARDS Network (ClinicalTrials.gov number NCT00281268) evaluating two types of catheters (PAC vs. CVC) and two fluid strategies (liberal vs. conservative) in the management of patients with ALI, with a total enrolment of 1001 patients. A liberal fluid strategy aimed at a central venous pressure (CVP) of 10–14 mmhg (by CVC) or pulmonary artery occlusion pressure (PAOC) of 14–18 mmHg (by PAC), while a conservative strategy aimed at a CVP<4 and PAOP<8 mmHg in the CVC and PAC arms. The trial's primary end-point was 60-day all-cause mortality.

To participate in the EA-PAC study, subjects had to be enrolled in FACTT and they or their proxies had to provide informed consent for the follow-up procedures. EA-PAC was approved by the NHLBI ARDS Network steering committee and the institutional review boards (IRBs) of all participating sites. Coordinating center approval for the long-term follow-up study was obtained from the University of Pittsburgh IRB. FACTT was monitored by a data safety and monitoring board and the long-term follow-up was additionally monitored by an NHLBI-funded advisory board.

### Long-Term Outcomes

For those consenting to long-term follow-up, we conducted interviews blinded to treatment assignment at 2, 6, 9 and 12 months. We administered the Health Utilities Index Mark 2 (HUI-2) questionnaire to assess health utility.[11] Where possible, we spoke with the subject.[12] One-year vital status and date of post-discharge death when applicable were ascertained from hospital records, interview, and search of the National Death Index.

### Costs and resource use

We obtained the detailed hospital bill for the initial hospitalization and calculated costs using the Medicare year-specific hospital and department-specific cost-to-charge ratio (www.cms.hhs.gov/CostReports). We assessed post-discharge healthcare costs by collecting information during follow-up interviews on re-hospitalizations, physician and emergency department visits, medications, and ancillary support. The cost for post-discharge use was calculated by multiplying unit cost weights with units of resource use, where cost weights were estimated from existing data (Table 1). All costs were then updated and expressed in 2010 US dollars using the U.S. Census consumer price index.

**Table 1:** Cost weight in the assessment of post-discharge resource use.

	Cost weight	Justification
Home oxygen	First day = 600$ +6.50/day other days	www.vgmdclink.com/pdffiles/MorrisonOxygenCostStudyJune2006.pdf
Home Help	$100/day	1999 CMS cost report, indexed to 2009
Rehab	$800/day	1999 CMS cost report, indexed to 2009
Skilled nursing facility (vent)	$1500/day	1999 CMS cost report, indexed to 2009
ED visits	$800/day	1999 CMS cost report, indexed to 2009
DR visits	$200/day	1999 CMS cost report, indexed to 2009
Rehospitalization	Total days*1500+ICU day*(2600–1500)+vent days*(2800–2600)	Cost of individual days obtained from State discharge database analysis^9^
Medications	$10/day	Estimated
Lost days of work	$200/day	Median US daily income

CMS - Center for Medicare and Medicaid Services.

### Data analysis

Non-parametric tests were used to compare continuous data between treatment of the PAC and CVC arms. Categorical data and proportions were compared by chi-square. Survival was evaluated by Kaplan-Meier analysis and groups compared by log-rank test. W conducted a longitudinal analysis for the repeatedly measured utilities using the Generalized Estimating Equations (GEE) method. Linear and quadratic trends over time were examined in the linear regression models. All analyses were conducted using SAS 9.0 software (SAS Institute, Cary NC).

### Cost-effectiveness

We used the U.S. Public Health Service Panel on Cost-effectiveness in Health and Medicine recommendations and the American Thoracic Society guidelines to conduct the economic analysis from a societal perspective.[10], [13] For each subject enrolled in EA-PAC, we constructed: i.) a stream of costs to one year post-enrolment based on calculated hospital costs, post-discharge direct medical costs, and lost wages ; ii.) a stream of projected lifetime costs based on projected survival (*infra vide*); iii.) effect at one year post-enrolment, defined as duration of survival multiplied by the area under the utility curve over the first year using utility data from interval interviews, and; iv.) projected lifetime accrual of quality-adjusted life-years(QALYs).

We calculated an incremental cost-effectiveness ratio (ICER) at one year based on data collected to one year. We also generated a reference case as follows. We estimated life expectancy for each subject alive at one year post-enrolment based on the year 2006 age-, race/ethnicity-, and sex-specific U.S. life tables. We assigned an average utility of 0.6 beyond one year based on prior studies.[8], [14] We assigned costs beyond one year to each subject's projected remaining years of life based on the National Expenditure Medical Survey (www.meps.ahrq.gov) and used a 3% annual discount rate for costs and effects.[15] The ICER comparing PAC and CVC was obtained as the ratio of the difference in cost to the difference in effect per enrolled subject and a Monte Carlo sensitivity analysis (5000 simulated trials) was conducted to reflect uncertainties in costs and effects. We calculated the overall ICER, the 95% confidence ellipse around this estimate, and the probability of the ICER falling below $50k/QALY and below $100k/QALY.[16] We generated a cost-effectiveness acceptability curve for the reference case. We generated model estimates for several a priori defined subgroups: sex, age, ethnicity, fluid strategy, APACHE III score (< and >90), tidal volume (≤ and >6.9 cc/kg), time from diagnosis to initiation of study protocol (≤ and >21.5 h), and ARDS etiology. We conducted secondary sensitivity analyses to evaluate the impact on cost-effectiveness of expected survival, utility, estimated yearly cost of health care beyond the first year, physician reimbursements and discounting rate.

## Results

### Patients

**Figure 1:**
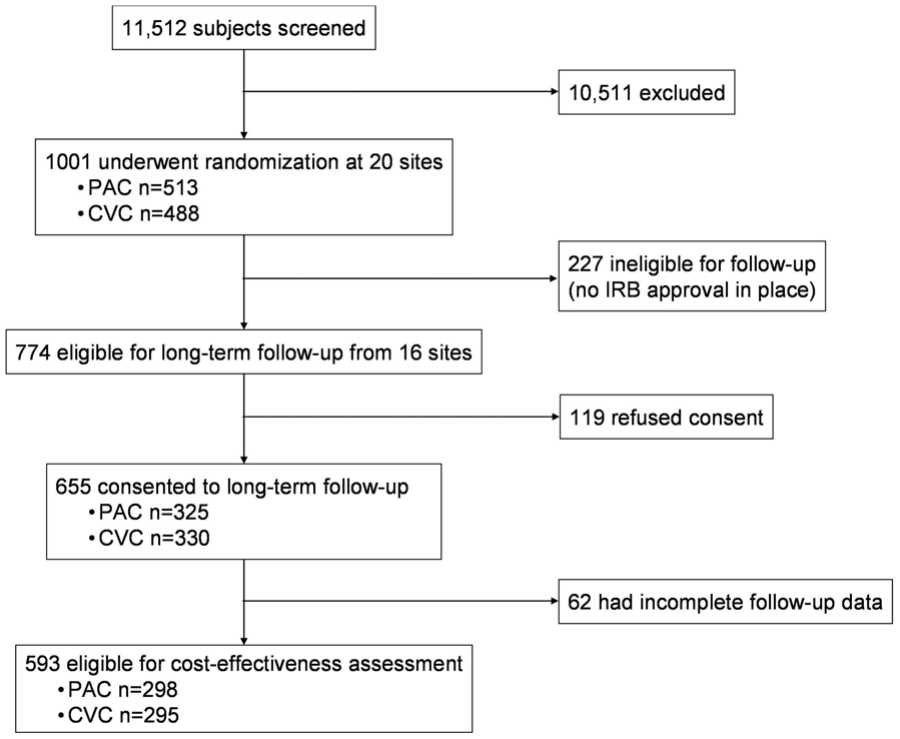
Quorum chart of the EA-PAC cohort.

There were 10,511 patients screened for enrolment at 20 North American centers between June 8, 2000, and October 3, 2005. Of the 1001 subjects analyzed in FACTT, 774 (77%) were enrolled at sites and during time periods for which IRB approval was granted for the EA-PAC study. Of these 774 subjects, 655 (85%) agreed to participate and one-year outcome was available in 593 (Figure 1). Subjects who participated in EA-PAC were generally similar to those who did not, but were less likely to have HIV infection or AIDS, more likely to be non-Hispanic whites, and more likely to have a lower cardiac index and be receiving smaller tidal volumes at enrolment (Table 2). There were no differences at baseline in the EA-PAC cohort between subjects assigned to PAC or CVC treatment other than a slightly longer time to initiation of protocol and lower proportion of HIV infection or AIDS in PAC subjects (Table 3).

**Table 2:** Comparison of EA-PAC and FACTT Cohort.

	EA-PAC Cohort	FACTT Cohort not in EA-PAC	Total FACTT cohort	p-value
N	655	346	1001	
Age, mean (SD)	50.1 (16.0)	49.1 (16.7)	49.8 (16.0)	.34
Sex, female, n (%)	308 (47%)	158 (49%)	466 (47%)	.74
Race, n (%)				<.001
White non-Hispanic	445 (68%)	196 (57%)	641 (64%)	
Black non-Hispanic	136 (21%)	81 (23%)	217 (22%)	
Other	74 (11%)	68 (20%)	142 (14%)	
Primary Lung injury, n (%)				.12
Pneumonia	299 (46%)	172 (50%)	471 (47%)	
Sepsis	145 (22%)	88 (26%)	233 (24%)	
Aspiration	103 (16%)	46 (13%)	149 (15%)	
Trauma	50 (8%)	24 (7%)	74 (7%)	
Multiple transfusions	6 (1%)	3 (1%)	9 (1%)	
Other	46 (7%)	11 (3%)	57 (6%)	.
Co-existing Conditions, n (%)				
None	444 (68%)	216 (63%)	660 (66%)	.11
Diabetes	110 (17%)	63 (19%)	173 (18%)	.66
HIV Infection or AIDS	36 (6%)	35 (10%)	71 (7%)	.01
Cirrhosis	24 (4%)	9 (3%)	33 (3%)	.46
Solid Tumors	12 (2%)	3 (1%)	15 (2%)	.28
Leukemia	12 (2%)	10 (3%)	22 (2%)	.37
Lymphoma	6 (1%)	7 (2%)	13 (1%)	.16
Immunosuppression	51 (8%)	27 (8%)	78 (8%)	1.0
Apache III score, mean (SD)	93 (31)	95 (30)	94 (31)	.51
Medical ICU (%)	421 (64%)	242 (70%)	663 (66%)	.07
Cardiorespiratory variables, mean (SD)				
MAP (mm Hg)	77 (14)	77 (14)	77 (14)	.61
CI (liters/min/m2)	4.1 (1.4)	4.4 (1.5)	4.2 (1.4)	.04
Vasopressor use, n (%)	248 (39%)	150 (44%)	398 (40%)	.10
Pre-randomization fluid balance (mL)	2875 (3590)	2553 (3417)	2764 (3533)	.18
PaO2/FiO2	127 (57)	127 (62)	127 (58)	.86
Tidal volume (mL), mean (SD)	452 (99)	490(122)	466 (109)	<.0001
TV (mL/kg of PBW), mean (SD)	6.3 (2.7)	6.8 (3.1)	6.5 (2.9)	<.0001

SD - standard deviation; MAP - Mean arterial pressure; CI - Cardiac index; TV - Tidal volume.

**Table 3:** Baseline characteristics by treatment assignment for the EA-PAC cohort.

	PAC	CVC	p-value
N	325	330	
Age, mean (SD)	50 (16)	50 (16)	0.91
Sex, female, n (%)	153 (47)	155 (47)	0.98
Race, n (%)			0.04
White non-Hispanic	231 (71)	213 (65)	
Black non-Hispanic	63 (19)	73 (22)	
Hispanic	22 (7)	40 (12)	
Other	9 (3)	4 (1)	
Primary lung injury, n (%)			0.62
Pneumonia	153 (47)	146 (44)	
Sepsis	70 (22)	75 (23)	
Aspiration	46 (14)	57 (17)	
Trauma	29 (9)	21 (6)	
Multiple transfusions	2 (1)	4 (1)	
Other	25 (8)	27 (8)	
Co-existing conditions, n (%)			
None	217 (67)	227 (69)	0.58
Diabetes	56 (18)	54 (17)	0.85
HIV infection or AIDS	12 (4)	24 (8)	0.04
Cirrhosis	12 (4)	12 (4)	0.99
Solid tumors	5 (2)	7 (2)	0.56
Leukemia	8 (3)	4 (1)	0.25
Lymphoma	3 (1)	3 (1)	0.99
Immunosuppression	29 (9)	22 (7)	0.31
APACHE III score, mean (SD)	96 (31)	92 (31)	0.08
Medical ICU, n (%)	208 (64)	213 (65)	0.85
Cardiorespiratory variables, mean (SD)			
Mean arterial pressure, mm/Hg	78 (15)	77 (14)	0.50
Cardiac index, L/min/m2	4 (1.4)	N/A	N/A
Vasopressor use, n (%)	132 (42)	116 (36)	0.13
Pre-randomization fluid balance, mL	2962 (3730)	2790 (3451)	0.61
PaO2:FiO2 ratio	126 (58)	128 (56)	0.56
Tidal volume index, ml/kg, mean (SD)	7.13 (1.54)	7.19 (1.53)	0.65
Time from diagnosis to protocol initiation, mins, mean (SD)	1523 (912)	1365 (815)	0.04

SD =  standard deviation; PAC  =  pulmonary artery catheter; CVC  =  central venous catheter.

### Clinical outcomes

**Figure 2:**
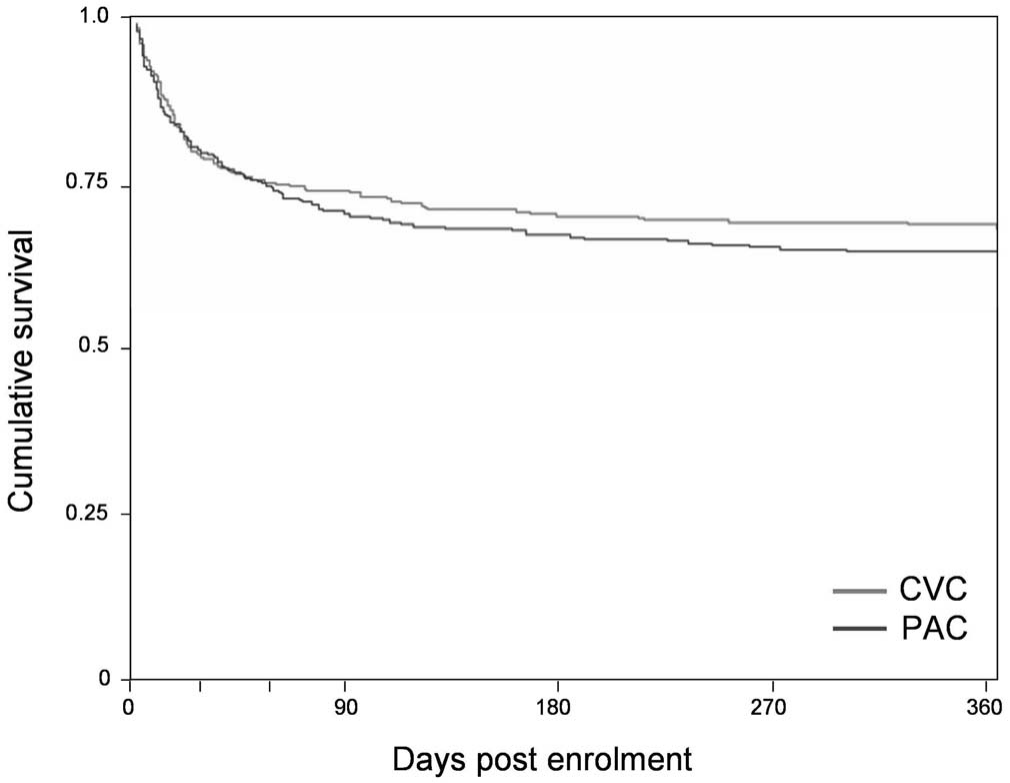
Survival by treatment arm. Trends seen in the FACTT trial persist to one year of follow-up. Although patients with CVC have higher cumulative survival, the difference is not significant (p = 0.33, log-rank).

**Figure 3:**
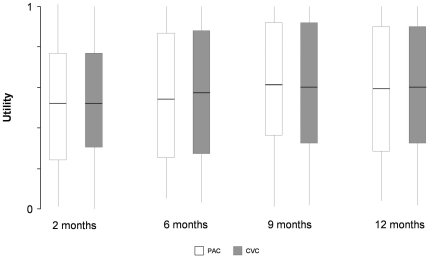
Utility by treatment arm. Median Health-related quality-of-life, measured by the Health Utilities Index, is uniformly low in the EA-PAC cohort, although the inter-quartile range is wide and individual values spread the entire 0–1 interval. Utilities are lowest at 90 days and improved by 9 months. Subjects assigned to the PAC were no different than those assigned to the CVC.

Mortality estimates from the Kaplan-Meier analysis increased from 26.2% and 25.1% at two months in the PAC and CVC groups to 35.6% and 31.9% at one year. Cumulative survival at one year was similar in the PAC and CVC arms (p = 0.33) (Figure 2). Of the 531 EA-PAC subjects discharged alive, we conducted at least one interview with 429 (80.7%) subjects or their proxies. Fifty subjects had only 1 interview, 47 had 2 interviews, 87 had 3 interviews and 245 completed all 4 interviews. Of 1385 interviews, 344 (25%) were obtained from proxies. Median quality of life was low throughout follow-up but did improve from two months (0.47 and 0.51, p = 0.47) to one year (0.61 and 0.66, p = 0.49) (p = 0.004 for trend over time), with the improvement plateauing at 9 months (p = 0.04 for quadratic trend). There were no differences in quality of life either at any time point or overall between the PAC and CVC arms (Figure 3).

### Healthcare costs

**Figure 4:**
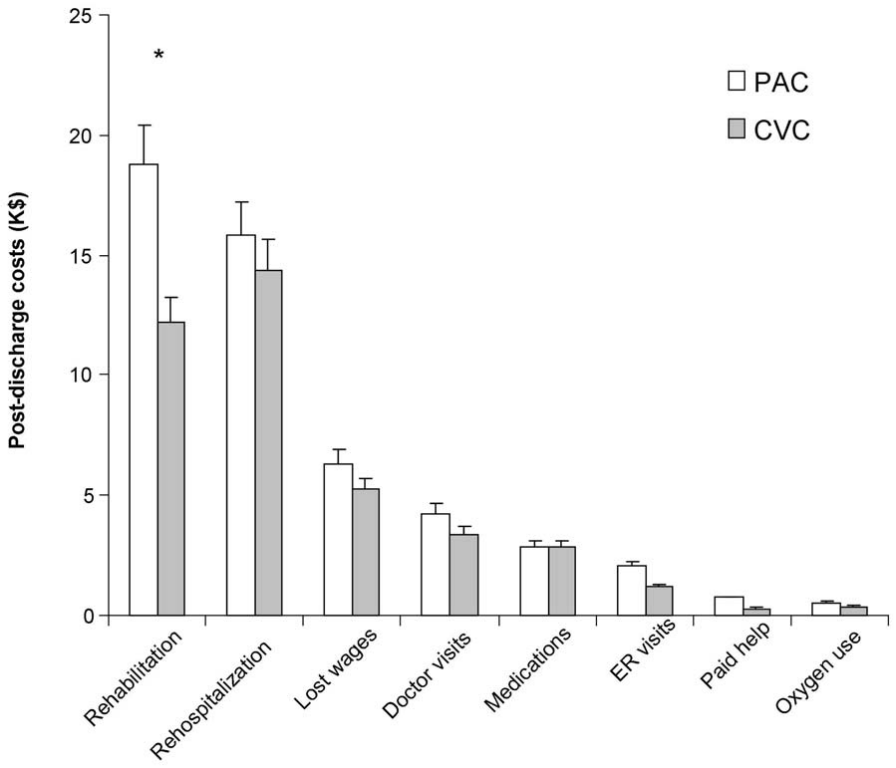
Post-discharge resource use. Overall post-discharge costs were significantly higher in patients assigned to the PAC. There was a trend in most categories of costs favoring CVC, but only post-discharge rehabilitation costs were significantly different. Of note, the difference was most apparent at the 9 and 12 month follow-up point (data not shown).

Overall, subjects incurred long and expensive hospitalizations. Hospital costs were available for 633 (97%) of the 655 EA-PAC subjects and were similar for the PAC (n = 312) and CVC (n = 321) groups ($96.8±86.8k vs. $89.2±74.5k, p = 0.38). Length of hospital stay post-enrolment was also similar (24.4±19.2 vs. 23.8±19.8 days, p = 0.48) (Table 4). However, for patients discharged alive costs to one year were higher in those receiving a PAC ($61.1±80.6k vs. 45.4±65.7) (p = 0.03). Although differences in post-discharge costs calculated from resource use (Table 5) did not reach statistical significance, they tended to be consistently higher in the PAC arm (Figure 4).

**Table 4:** Post-discharge resource use.

	PAC (n = 210)	CVC (n = 219)	All (n = 429)	p-value
Home oxygen use				
Number of subjects, n (%)	66 (31.4%)	64 (29.2%)	130 (30.3%)	.62
Duration days, Mean + SD (median)	90.4±109.4 (31.5)	86.8±108.7 (30.0)	88.6±108.7 (30.0)	.94
Rehospitalizations				
Number of subjects, n (%)	101 (48.1%)	93 (42.5%)	194 (45.2%)	.24
Times of re-hospitalization, Mean ± SD(median)	3.1±4.0 (2.0)	2.5±2.7 (1.0)	2.8±3.4 (2.0)	.32
LOS of re-hospitalization, Mean ± SD (median)	20.2±28.5 (9.5)	17.7±23.0 (8.0)	19.0±26.0 (9.0)	.71
Post-discharge rehabilitation				
Number of subjects, n (%)	72 (34.3%)	64 (29.2%)	136 (31.7%)	.26
Duration days, Mean ± SD (median)	72.9±94.3 (23.0)	54.0±68.4 (21.5)	64.0±83.4 (22.0)	.67
Post-discharge ED visits				
Number of subjects, n (%)	123 (58.6%)	116 (53.2%)	239 (55.8%)	.26
Number of ED visits, Mean ± SD (median)	4.3±4.5 (2.0)	3.1±3.1 (2.0)	3.7±3.9 (2.0)	.06
Post-discharge doctors' visit				
Number of subjects, n (%)	203 (96.7%)	214 (98.2%)	417 (97.4%)	.33
Number of Doctor visits, Mean ± SD (median)	20.3±22.0 (13.0)	19.4±30.6 (12.0)	19.9±26.7 (13.0)	.10
Post-discharge medications				
Number of subjects, n (%)	185 (88.1%)	201 (91.8%)	386 (90.0%)	.20
Number of Medications, Mean ± SD (median)	18.6±15.9 (15.0)	17.7±15.9 (13.0)	18.1±15.9 (14.0)	.44

SD - standard deviation; PAC - pulmonary artery catheter; CVC - central venous catheter; NS - non-significant.

**Table 5:** Estimates of mean costs and effects from Monte Carlo simulations.

	PAC, mean (SD)	CVC, mean (SD)	Incremental
Costs (thousand $)			
Hospital cost*	93.3 (4.8)	84.4 (3.7)	8.9
Post-discharge cost to one yr*	46.7 (4.7)	35.2 (3.6)	11.5
Lifetime cost	191.1 (8.5)	176.7 (7.1)	14.4
Effects			
Life expectancy	19.0 (1.0)	20.1 (1.0)	−1.0
QALY	4.54 (0.21)	4.83 (0.21)	−0.30

SD- standard deviation; PAC – Pulmonary artery catheter; CVC – Central venous catheter; QALY – Quality adjusted life-years.

* With replacement of outliers exceeding the 95th percentile.

### Cost-effectiveness

**Figure 5:**
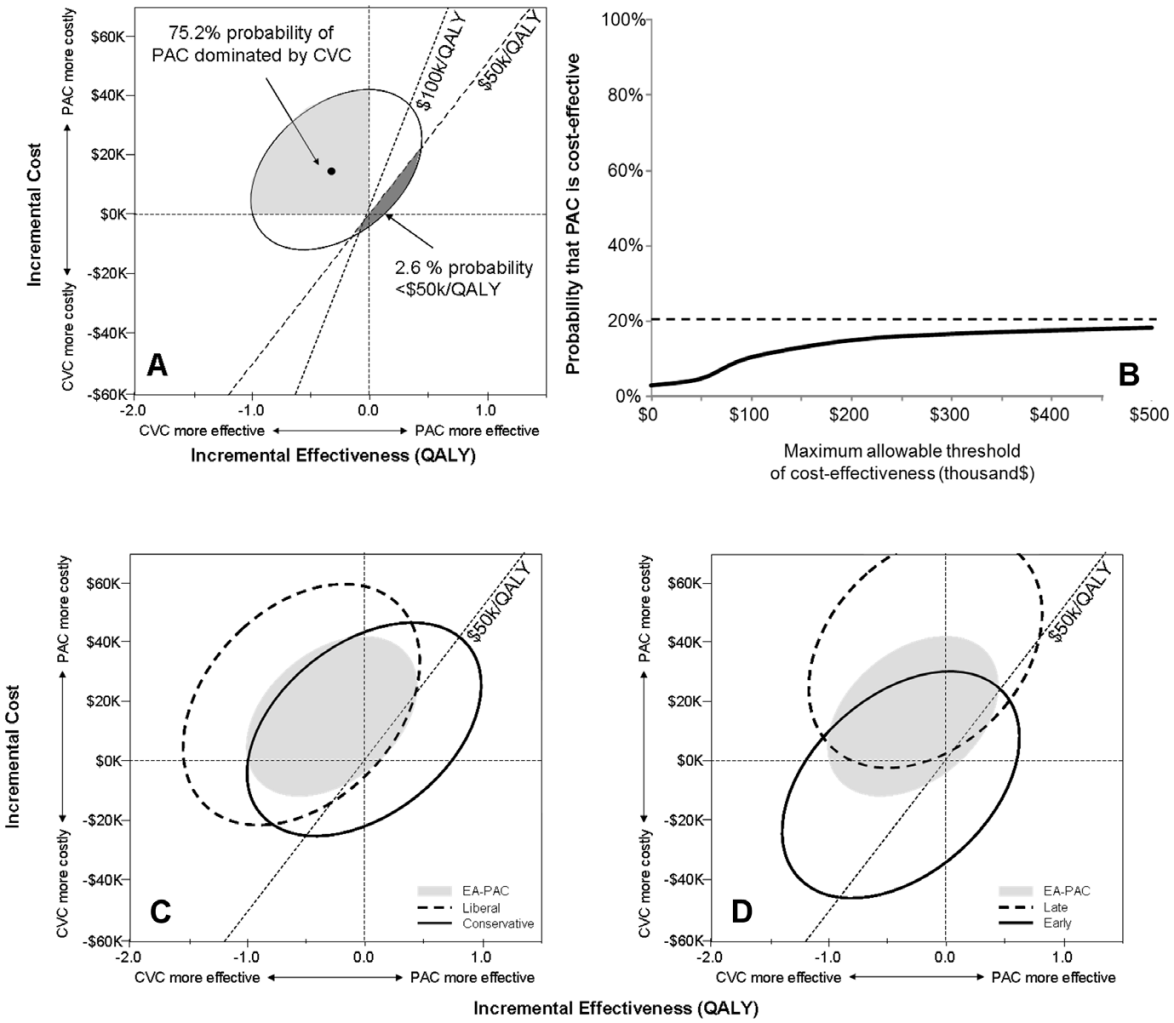
Cost-effectiveness of the Pulmonary Artery Catheter. The mean estimate of incremental costs and effects suggest that the PAC is both more expensive and less effective (panel A). The 95% confidence ellipse only marginally dips below the 50 k/QALY willingness-to-pay threshold with the vast majority of trials agreeing with the mean estimate that the PAC is an inferior strategy. The corresponding cost-effectiveness acceptability curve conveys the probability of the PAC to be cost-effective at various willingness-to-pay thresholds (Panel B, x-axis). Even if willingness-to-pay was unlimited, there is only a 20.8% probability of the PAC to be cost effective (Panel B, dotted line). The PAC displays a better cost-effectiveness profile in subjects treated with a conservative fluid strategy (panel C) than those receiving a liberal strategy. A similar trend is seen in subjects where the study protocol was instituted early after enrolment (panel D). Yet, for both subgroups, there was a high probability for the PAC to be an ineffective strategy.

At one year post-enrolment, average total healthcare costs were $130.4±112.6 vs. $115.4±98.8k for the PAC and CVC groups (p = 0.22), while average accrued quality adjusted days were 120.3±110.3 vs. 127.5±109.7 days (p = 0.50). Estimates of mean hospital costs (93.3±4.8 vs. 84.4±3.7), post-discharge costs to one year (46.7±4.7 vs. 35.2±3.6), lifetime costs after the first year, and total health care costs (191.1±8.5 vs. 176.7±7.1) obtained from Monte Carlo trials were higher for the PAC cohort (Table 6). Subjects receiving a PAC had a slightly shorter life-expectancy (19.0±1.0 vs. 20.1±1.0 years) and fewer QALYs (4.54±0.21 vs. 4.83±0.21 QALYs) than those receiving a CVC (Table 5). Consequently, the mean of all simulated trials suggested that PAC use was both more expensive by $14.4 and less effective by 0.30 QALY than CVC use (Table 6). There was a 75.2% probability that PAC use was both less effective and more expensive, and 97.4% and 94.2% probabilities that PAC use exceeded the $50k/QALY and $100k/QALY willingness-to-pay thresholds (Figure 5, panel A). A cost-effectiveness acceptability curve suggests that a maximum of only 20.8% of simulations could ever be acceptable, even if unlimited financial resources were available (PAC use >$1M/QALY) (Figure 5, Panel B).

**Table 6:** Mean estimates of costs and effects for EA-PAC subgroups.

	N	Incremental Costs ($k)	Incremental Effects (QALY)	% trials with PAC inferior	% trials with PAC < $50k/QALY
All	593	14.4	−0.30	75.2	2.6
Age group					
<45	213	10.4	−0.11	35.1	24.8
45–64	256	9.4	−0.69	68.5	1.9
>64	124	33.5	−0.11	53.1	6.1
Sex					
Female	288	18.3	−0.21	57.8	7.9
Male	305	11.2	−0.39	61.1	7.6
Ethnicity					
White non-Hispanics	408	0.9	−0.44	61.1	6.2
Black non-Hispanics	120	50.5	0.06	44.1	7.8
Hispanics	54	−21.7	−0.49	15.3	48.3
Fluid strategy					
Liberal	300	18.7	−0.58	80.1	2.0
Conservative	293	9.7	−.03	34.7	29.0
APACHE III score					
>90	288	27.8	0.44	13.9	40.1
≤90	285	2.7	−0.62	53.8	4.5
Delay to initiation of protocol (hours)					
≤21.5	298	−8.8	−0.40	20.8	29.1
>21.5	300	36.5	−0.18	66.2	1.7
Tidal volume (cc/kg)					
>6.9	268	11.5	−0.61	67.1	2.7
≤6.9	254	18.0	0.29	21.4	44.4
Diagnosis					
Sepsis	137	18.7	−0.40	70.3	4.0
Pneumonia	271	2.1	−0.44	38.2	24.6
Aspiration	90	37.9	−0.25	55.9	8.3

PAC – Pulmonary artery catheter; QALY – quality-adjusted life-year.

### Sensitivity analyses

The cost-effectiveness estimates were robust to varying assumptions regarding costs and effects. Decreasing life expectancy to half that of an age-, race-, and sex-matched cohort marginally widened incremental costs to $16.6k (+15%; where a positive value indicates that the PAC is more costly), while incremental benefit was also lower at −0.21 QALY (−30%; where a negative value indicates that the PAC is less effective). Increasing or reducing the projected utilities beyond one year by 25% changed the estimated decrement with PAC use of −0.30 QALYs per subject to −0.35 (+17%) and −0.24 (−20%). Increasing the annual discount rate to 5% widened incremental costs slightly to 15.9 k (+10%), while narrowing incremental QALYs to −0.19 (−37%). Halving to doubling annual healthcare costs beyond one year resulted in incremental costs with PAC use of $18.4k (+28%) and $10.8k (−25%). Halving to doubling physician reimbursements had minimal impact on incremental costs. No combination of factors produced a mean PAC ICER better than $50k/QALY.

### Subgroup analyses

The probability that PAC use is cost-effective varied by subgroup. More favorable profiles were seen in patients who were treated with a conservative fluid strategy (Figure 5, panel C), had higher APACHE III scores, were ventilated with low tidal volumes, were younger, were of Hispanic descent, suffered from pneumonia as the underlying diagnosis, and were treated earlier (Figure 5, panel D and Table 6). Yet none of the subgroups examined had >50% of the simulations with ICER <$50k/QALY.

## Discussion

Consistent with prior series of subjects with ALI, the patients enrolled in this trial incurred significant post-discharge morbidity and high costs of care.[6], [8], [17] Both hospital and post-discharge costs were higher in PAC subjects, in association with an increased incidence of rehospitalization and rehabilitative care, although differences in post-discharge costs did not reach statistical significance. There was no evidence that management with a PAC had any salutary benefits on these outcomes. Indeed, subjects managed with the PAC incurred higher costs and perhaps fared worse, a finding that extends results of the short-term efficacy trial. These findings were robust to multiple sensitivity analyses and were broadly consistent across a variety of subgroups. Thus, with respect to conventional management with a CVC, the FACTT trial suggests use of the PAC in ALI is ineffective, more expensive, and associated with increased long-term morbidity and costs after hospital discharge.

We tested PAC use in a setting where data from the catheter drove explicit protocolized instructions for titration of intravenous fluids, diuretics, and vasoactive agents. Our results were similar regardless of whether the protocols advocated liberal or conservative fluid management. A potential criticism of our findings is that they may not be generalizable to the setting where expert clinicians make individualized decisions based on hemodynamic data. However, a recent British study randomized critically ill subjects to monitoring with the PAC where all treatment decisions remained at the discretion of the treating clinician. Despite the differences in study design, the British study also found that PAC use yielded no cost savings and potentially worse outcomes.[18] The authors further concluded that it would be cost-effective to withdraw PACs from all British ICUs.

One and two-year follow-ups of patients with ALI suggest that burden of chronic illness, rather than any acute care sequence of event, is the key determinant of long-term morbidity.[6], [7] The increase in resource use and post-discharge costs observed in patients treated with the PAC does not contradict such observations. These observations would suggest that the PAC alters the course of an existing chronic illness or gives rise to an additional burden unaccounted for by what is traditionally perceived as a chronic illness. For example, it known that increased inflammation is associated with increased risk of acute cardiovascular events[19] and that residual inflammatory burden at hospital discharge is linked to post-discharge mortality.[20], [21] Whether the PAC itself or co-interventions promote increased inflammatory burden remains to be investigated.

Although use of the PAC has declined in the US, several hundreds of thousands of patients still receive it annually.[5] This ongoing use despite failure of the prior clinical trials to demonstrate benefit perhaps reflects a high physician comfort level with the information provided by the PAC. Preference for the PAC could be bolstered by the fact that the negative trials did not conclusively demonstrate harm and could not exclude the possibility of non-mortal benefits. However, in this era of escalating healthcare costs it is hard to justify continued broad use of an intervention with such a poor cost-effectiveness profile. Rather, PAC use should probably be curtailed to select instances where specific diagnostic information is required, such as in the evaluation of suspected pulmonary hypertension.

As with prior studies on the effectiveness of the PAC, true differences or lack thereof between treatment and control groups might be veiled by unmeasured confounders. However, adding cost of care information provides further evidence of the robustness of a recommendation of highly discriminatory use of the PAC. We performed four questionnaires in the first year. Longitudinal trends observed in the recovery of utilities could not have been detected with a sparser schedule. Yet our study also suggests that a 3 month and 9 month follow-up would capture the nadir and recovery of indices of quality of life while minimizing burden on both subjects and researchers. Approximately a quarter of questionnaires were filled with proxies, which may not be particularly reliable for subjective information relating to quality of life.[12], [22] Our estimates are however quite robust to uncertainty in quality of life of survivors and in fact to post-discharge costs derived for the follow-up questionnaire. The willingness-to-pay thresholds we used have been proposed several years ago and may be too low today.[16], [23] However, the conclusions did not change using more generous thresholds. We did not provide a formal ICER for a PAC-based strategy because such a ratio is only informative (and sensible) for effective strategies.[24] Follow-up beyond one year would have been desirable to determine whether the high resource use and mortality in the first year continued. Our study was conducted from a societal perspective with a reference case that includes a lifetime horizon. From a healthcare provider's perspective, added hospital costs of the index hospitalization with the PAC would likely not effect reimbursements, while post-discharge costs may likely be cost-neutral, resulting also in an unfavorable cost-effectiveness profile.

The use of the PAC in patients with ALI appears to increase costs of care, produces no short or long-term benefit, and is associated with trends towards worse outcome in some long-term measures. This unfavorable cost-effectiveness profile does not justify routine use of the PAC in patients with ALI and broadens the conclusions of a large short-term efficacy trial.
